# Clinical benefits of β-glucan supplementation in children: a review

**DOI:** 10.1007/s44187-022-00038-0

**Published:** 2022-12-27

**Authors:** Rachana Bhoite, Vinita Satyavrat, Manasa Premasudha Sadananda

**Affiliations:** Dr Reddy’s Laboratories Pvt Ltd, Ameerpet, Hyderabad, India

**Keywords:** β-glucan, Immunity, Pediatric, Dietary supplements

## Abstract

Malnutrition is a global concern since it affects 130 million children under the age of 5 worldwide. The child’s immunity is brutally compromised, making them susceptible to various diseases and infections, leading to a high mortality rate. Multiple strategies have been implemented to strengthen immunity in children with compromised immunity, such as rendering a balanced diet, fortifying food, dietary supplements, and introducing potential natural dietary fibers to maintain good nutrition status, such as β-glucan. A class of biologically active polysaccharides, sourced from yeast, mushroom, bacteria, and cereals with versatile immunomodulatory benefits that potentially primes the host immune system, drives several metabolic health effects, increases infection resistance, shields against the damaging effects of stress, and maintains healthy energy levels. This review focuses on the capabilities, underlying mechanisms, immune-stimulating potency, and clinically meaningful shreds of evidence to substantiate the multiple health benefits of β-glucan in children. Although, more clinical studies are required, current findings confirms the various biological response modifying abilities of β-glucan that may notably aid in the development of a strong immune system in children for their overall health and wellbeing.

## Introduction

Preschooler’s common concerns include delayed growth, being underweight, overweight, feeding delays, oral-motor problems, allergies, rashes, gut (constipation and diarrhea), and respiratory health concerns. Furthermore, altered feeding interactions, medication/nutrient interactions, altered energy and nutrient needs (inborn errors of metabolism), and poor or excessive appetite are frequently seen in children [1, 2]. An increasing body of evidence indicates a link between malnutrition and reduced immunity and poor cognitive ability in children, leading to inflammation, chronic diarrhea, and acute upper respiratory tract infection affecting the child’s overall health and general well-being [3]. Children suffering from these conditions are underweight due to inadequate nutritional intake, a lack of appetite, lack of sleep, and being undernourished [4].

In 2021, the World Health Organization (WHO) defined malnutrition as forms that include undernutrition (wasting, stunting, underweight), inadequate vitamins or minerals, overweight, obesity, and resulting diet-related non-communicable diseases [5]. Long-term malnutrition affects children's growth, resulting in stunting, affecting more than 30% of children aged five years in low and middle-income countries [6]. According to the WHO, 155 million children under the age of five are stunted, 52 million are underweight, 17 million are severely underweight, and 41 million are overweight or obese.

When a child consumes more calories than required and often from low-nutrient sources (i.e., non-nutrient-dense foods), they become obese or gain unhealthy weight [4]. In addition, childhood obesity induces adult diseases in children, such as high blood pressure, type 2 diabetes mellitus (T2DM), and heart disease [7]. Most undernourished children die from common infections like malaria, pneumonia, tuberculosis, and diarrhea, implying that mortality is linked to underlying immunodeficiency, even in mild cases of malnutrition. In undernourished/malnourished children, weakened immunity (defects in innate and adaptive immunity) have been consistently demonstrated to pose a higher occurance of experiencing frequent infections and severe symptoms [8].

### The impact of the low or weakened immune system in children

#### Susceptibility to infection and diseases

Infection and malnutrition have a bidirectional relationship. The specific type of pathogen and the interaction location can influence the sort of immune reaction that takes place and if it is idyllic. The stimulation and generation of immune cells like T lymphocytes and the synthesis of antigens and their chemical properties are required to initiate innate and adaptive immune responses; the associated DNA replication, RNA expression, protein synthesis, and secretion consume additional anabolic energy. As a result, the host's nutritional status significantly impacts the infection's outcome. Children who are malnourished are more susceptible to acquire gastrointestinal and respiratory infections caused by bacteria [9]. The innate immune response, particularly epithelial barriers and the mucosal immune response, are the first lines of defense against these infections. Protein-calorie malnutrition (PCM) significantly compromises the mucosal barrier in the gastrointestinal, respiratory, and urogenital tracts. Vitamin A deficiency, for example, causes the loss of mucus-producing cells [11]. As a compromised gut barrier allows the higher toxins to flow through, mucous membrane barrier defects play a crucial role in the pathogenesis of respiratory and gastrointestinal infections [10]. When comparing severely malnourished children to normal children, serum levels of C3 protein tend to be lower [9]. The lack of it leads to severe disability in leukocyte microbicidal capacity early in infection, especially for gram-negative organisms, because it complements the initial events in phagocytosis and microbial killing [11].

Underdeveloped immune systems and high exposure to microbes at schools/childcare facilities contribute to a higher risk of illness in children. Data suggests that the average child gets 6 to 8 episodes of cold annually due to their poorly developed immune system. Malnutrition lowers children's immunity, making them more susceptible to diseases [12]. The leading causes of death in children differ depending on their age. Children under five are particularly vulnerable to infectious communicable diseases such as malaria, pneumonia, diarrhea, HIV, and tuberculosis. Non-communicable diseases such as cardiovascular diseases, diabetes, cancer, poor mental health all these pose significant risks to older children [13, 14]. 

#### Respiratory infections

Respiratory infections, such as pneumonia, are most common in children between the ages of 24 and 36 months when their immune systems are still developing and first exposed to pathogens. As a result of the immune response elicited by respiratory infection, the demand for metabolically derived anabolic energy increases, resulting in poor nutritional status. Furthermore, a respiratory infection can result in the loss of vital protein and energy stores in the body. Energy expenditure increases during an immune response, while nutrient intake decreases in the infected host. Furthermore, loss of weight, reduced food intake and infection induced nitrogen excreation is linked to negative nitrogen [10]. After a fever is induced, a negative nitrogen balance occurs, increasing and lasting for days to weeks after the febrile phase. As a result, repeated respiratory infections, common in young children, may result in malnutrition [9].

##### Epidemiology of respiratory infections

Respiratory disease is commonly seen in infants and young children aged 5–12 years. More than 90% of respiratory-related childhood deaths occur globally. Pediatric tuberculosis is another respiratory infection with high morbidity and mortality [15]. The common cold is one of the most common illnesses in children that leads to several doctor's visits and missed school days each year more than any other illness. During the fall and winter seasons, children are more likely to catch colds, thus, their immunity must be boosted during such times [16]. Most children recover in 7–10 days, but those with weakened immune systems or asthma may develop severe illnesses like pneumonia [17]. Influenza causes significant morbidity in children, resulting in the hospitalization of approximately 870,000 children under the age of five every year worldwide [18].

Acute lower respiratory infections (both bacterial and viral) are among the leading causes of illness and death in children globally. Pneumonia (lung infection) kills nearly 1 million children under the age of five each year, accounting for 15% of all deaths in children under the age of five. Infections with respiratory syncytial virus and *Streptococcus pneumonia* account for about 25% and 18.3% of all episodes of severe respiratory infection in young children, respectively. The influenza virus is another infectious organism contributing to children's global burden of respiratory disease [18]. As the maternal immunoglobulin G (IgG) is lost after the first year of life and has only reserves of acquired immunity, the respiratory virus still circulates in the community. Attention should be paid to children with frequent or prolonged infections [19, 20]. Acute respiratory infections (ARIs) are among the conditions that causes high mortality and morbidity in children under five years of age, and its consultation and treatment being the most used health service globally. ARIs leads to about 30–50% of medical consultation and about 20–40% of hospitalization of children. The leading causes of being prone to acquiring respiratory tract infections are poverty, restricted family income, low parental education, lack of breastfeeding, and malnutrition [21].

Recurrent respiratory infections (RRIs) are a condition that is collectively observed in children with significant social and economic consequences globally. RRIs affects about 25% of children under one year and 6% in their first 6 years of life, thus being a habitual reason for pediatric medical visits. Even though it is a benign condition that will likely improve over time by age of 12, it significantly impacts their well-being and incurs high medical and social costs [22]. About 60% had at least three episodes of acute otitis media, 73% had at least three antibiotic treatments, and 21% were admitted to the hospital with an acute respiratory infection. By 24 months, 13% of children had developed asthma. In addition, children who developed recurrent infections often had early nasopharyngeal colonization with *Streptococcus pneumonia* [19].

##### Relationship of immunity and respiratory infection

Children are involved in many sports activities. Athletes with high-intensity training or training in a cold environment are more likely to get upper respiratory tract infections (URTIs). In highly trained athletes the immune response can be similar to complex conditions such as that with J or S shape dynamics. High intensity trainings changes the immune response, thus raising biological markers of immune system such as natural killer cells (NK cells), T cells, B cells and increasing infection susceptibility. As a result, athletes, who are generally considered healthier than the general population, are more prone to respiratory related issues due to a compromised immune system at the time of intense training [23–25]. A study on athletic children playing hockey revealed that the facilities where children practice any outdoor game are exposed to gases like carbon monoxide, nitrogen dioxide and monoxide that are produced by ice cleaning machines. Thus, resulting in increased inflammatory cells (granulocytes and macrophages) in the respiratory tract of the children as they inhale this air, leading to increased number of wheezing and rhinitis episodes in children [26].

#### Overall growth and development of children

Infections such as bronchitis, oral thrush, upper respiratory infections, cellulitis, conjunctivitis, diarrhea, and stomatitis have been linked to weight gain in young children in developing countries [27]. Micronutrient malnutrition caused by acute and chronic infections can impair linear growth [28, 29]. Infectious diseases can cause micronutrient deficiencies in one of five ways: reducing food intake (anorexia), impairing nutrient absorption, causing direct micronutrient losses, increasing metabolic requirements or catabolic losses, and impairing transport to target tissues (Although this requires further research). Even if nutrients are absorbed, they may be lost due to pathogenic infection that causes direct nutrient loss into the stool or urine [27, 30].

## Major strategies to improve immune function and health status of children

### Nutrition and diet

Nutrition supporting the immune cell functions and enabling them to initiate efficient responses against pathogens and resolving the pathogen responses quickly when necessary, and avoiding any underlying chronic inflammation would be the ideal nutrition for the best immunological outcomes [31]. Each stage of the body’s immune response is dependent on the presence of numerous micronutrients in the body. Consuming sufficient nutrients as part of a varied diet is necessary for the healthy functioning of all cells, including immune cells. Food containing vitamins, minerals and proteins essential for developing and operating immune cells [32].

### Fortification of food

Micronutrient malnutrion in children can be best avoided by feeding/eating a well balanced diet and to make sure the diet includes enough of each nutrient. Fortifying food posses the benefit of providing nutrients to a large population without altering eating habits. This strategy is easy on the pocket and constant consumption of fortified food helps keep the micronutrient availability in the body in check [33].

### Dietary supplementation

Micronutrients like vitamin A help regulate genes involved in growth, vision, immunity, haematopoiesis, and tissue differentiation [34]. Vitamin B6 aids in cell-mediated immunity, lymphocyte maturation and growth, and increase the T-lymphocytes. Ascorbic acid, a major antioxidant, in a less active state helps synthesize collagen, regulates neurotransmitter metabolism, cholesterol metabolism, fatty acid transport, is a cofactor of the metalloenzymes and helps in maintaining the iron and copper atoms [35]. Vitamin D elicits the antimicrobial peptide production from the immune system [36]. Vitamin E improves the overall immune function. Zinc helps restore intestinal immunity, and thymulin activity, increases the cytotoxicity of NK cells, and reduces the number of activated T-helper cells, similarly iron improves intracellular microbial killing and aids in cellular immunity. Selenium improves cell-mediated immunity, raises the T helper cells, and aids in the immune response to viruses [37].

Micronutrients like carbohydrates, are essential for an adequate immune response. Galactose-involving interactions play a critical role in the host's defences and are essential for immune cell responses [38, 39]. Immune cells like T lymphocytes and B lymphocytes, White blood cells like macrophages, and NK cells are activated by amino acids [40].

Apart from the micro and macronutrients, high intakes of dietary fiber or prebiotics are effective in lowering the risks of developing metabolic diseases such as diabetes, obesity, coronary heart disease, stroke, hypertension, and certain gastrointestinal diseases [41]. β-glucan exudes prebiotic activity that positively modulates the growth of the gut microbiomes thereby exerting various health benefits in pediatric health conditions [42]. Beta glucans derived from cereals and mushroom are associated with numerous health benefits that include lowering cholesterol and improving heart health and even enhancing and supporting the immune system. Although currently, yeast-derived beta glucans (*Saccharomyces cerevisiae*) has been proved to have a well based immune-supporting properties [43].

## β-glucans

Dietary fibers such as β-glucans are soluble fibers that come from the cell walls of bacteria, fungi (including mushrooms), yeasts, seaweeds, and cereals (rice, oat and barley). They are a group of heterogenous non starch polysaccharides consisting of D-glucose monomers linked by glucosidic bonds. They boost the fiber content of food products and magnifiy the benefits [44].

### Sources

**Fig. 1:**
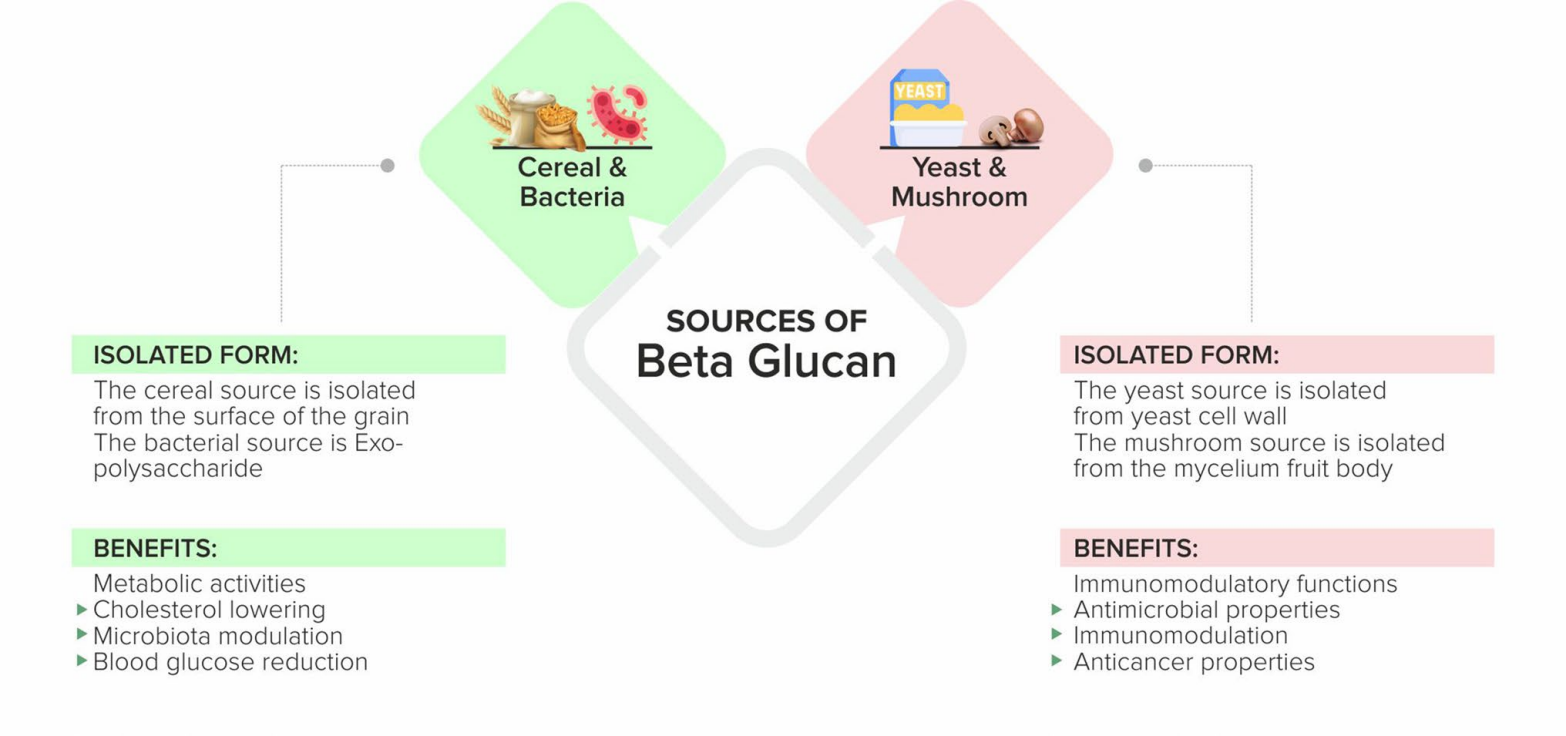
Sources of β-glucan. Adapted from [84]

β-glucans are sourced from namely two divisions cereal and non-cereal (Fig. 1). Cereal β-glucan usually has 1,3-1,4 glycosidic linkages without 1,6 bonds or branching. They are usually found in the cells of the plant seeds and the cell wall of the endospores such as oat, barley, wheat and rice. Non-cereal β-glucans include yeast, fungi, algae, and bacteria [45]. Unlike grain β-glucans, non-cereal β-glucans, such as the fungal source, differ between species regarding branching and distribution. For example, Curdlan, an agrobacterium sourced β-glucan, has no side branching, just a β-d glucan backbone [46]. The source of yeast derived β-glucan is of utmost importance, although both are derived from β-1,3/1,6 glucan of *Saccharomyces cerevisiae* that originates from either baker’s yeast or brewer’s yeast. The immune modulating abilities of the beta glucan extracted from baker’s yeast and brewer’s yeast differ due to the difference in its molecular pattern. The baker’s β-glucan imparts immune modulating properties but the benefits vary due to different strains. A unique yeast β-glucan (Wellmune) has extracted β-glucan from the cell wall of proprietary strain baker’s yeast and can be used by food, beverage and supplement brands to enhance or create products to support the immune health of all ages [43].

The dietary fiber is also distinguished through its solubility [47]. Higher molecular weight β-glucan is more effective than lower molecular weight β-glucan in cereal β-glucan. The diversity of β-glucan makes it versatile in different areas, such as alleviating respiratory illnesses to improving fatigue and weight loss. Non-cereal β-glucans, majorly fungal and yeast, have more pronounced immunomodulatory, anti-cancer properties and are a significant area of interest [48–50].

### Role of β-glucan as biological response modifier

#### Immunity

**Fig. 2:**
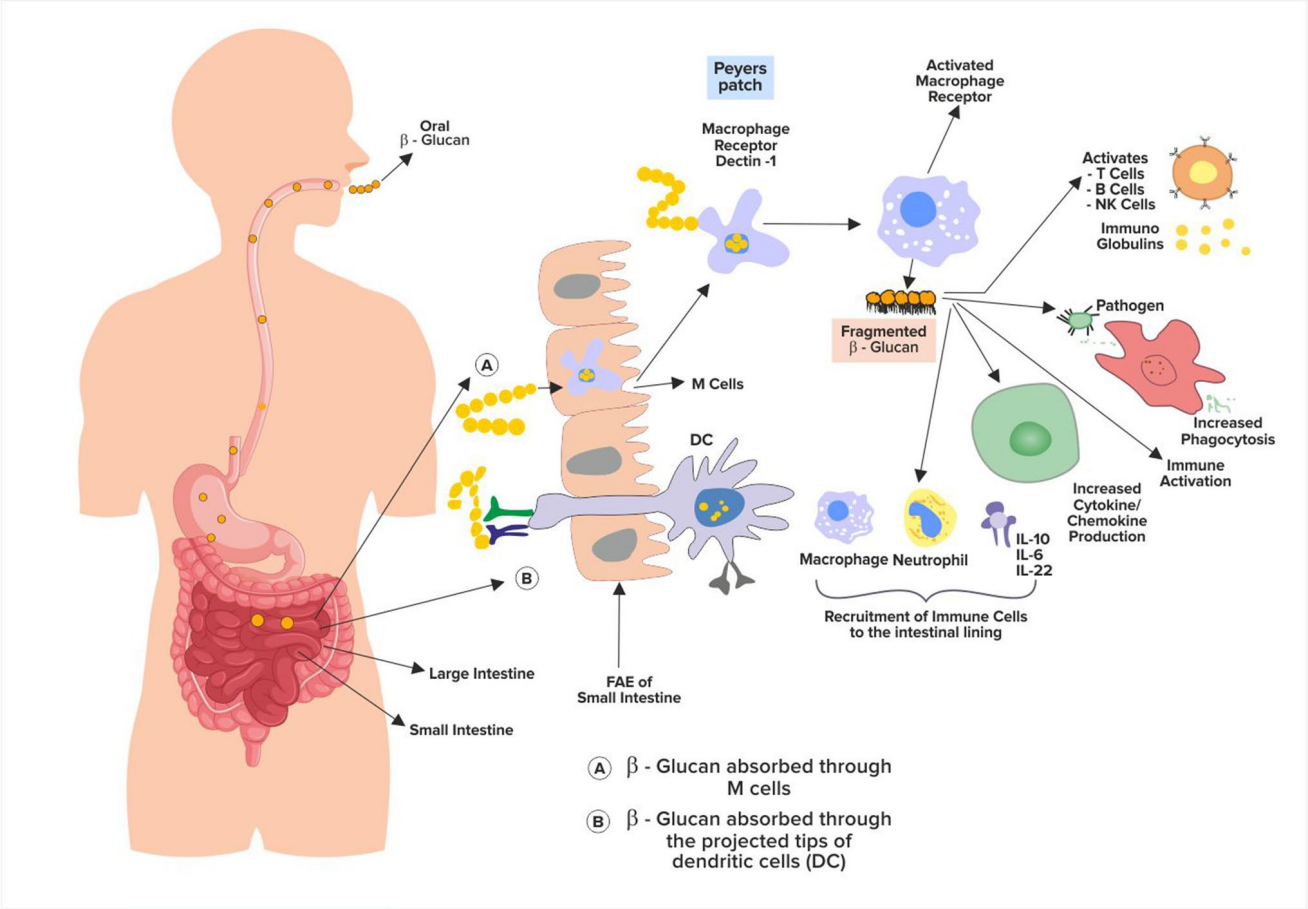
Mechanism of β-glucan in the body

β-glucans that are sourced from yeast or fungi are majorly recognized for their immunomodulatory effects. However, other sources may have similar properties but may be less potent compared to these sources [47]. The increased degradation of purine nucleotides such as adenosine and guanosine by β-glucan has been reported to increase immunity in infants [51]. β-glucans activate innate and adaptive mechanisms and cells, stimulate the neutrophils and macrophages' activities via the surface receptor, enhance phagocytic actions, provide support to the function of NK cells, modulate the role of antigen-presenting cells (e.g., langerhans cells), influences the production of cytokines and chemokines, promotes antigen presentation, creates a conditioning environment for Th1 lymphocytes, and modulates the antibody production [52]. The first β-glucan mediating step might be immunostimulation, i.e., binding the dietary fiber to the specific receptors in macrophages and dendritic cells, which induces the production of various cytokines and thus activates other immune cells such as T and B cells. Systemic immunostimulation might be the key to averting the growth of cancer cells and infective microorganisms in the host (Fig. 2). Dectin-1 and toll like receptor TLRs, which are β-glucan receptors in the macrophage and dendritic cells, are the leading key players in recognizing β-glucan. However, the relation of exact signaling pathways downstream from the respective receptors is still unclear [53].

Barley β-glucans have many metabolic benefits associated with the SCFAs production in the gut by the gut microbiota and the promotion of gut hormone secretion. It is beneficial in increasing plasma Peptide YY PYY and glucagon like peptide-1 GLP-1, which suppress appetite and promote glucose-dependent insulin secretion. The promotion of gut hormone secretion from enteroendocrine cells by SCFAs such as acetate, propionate, and butyrate are responsible for suppressing food intake and improvement in insulin sensitivity induced by barley flour diets. The action of SCFA-stimulated gut hormone secretion likely involves the SCFA receptors GPR41 and GPR43 [54–56].

The mechanism of action of Wellmune is similar to that of baker’s yeast β-glucan. Wellmune is well absorbed by the gut cells and imparts the immune benefits of the glucan. The said β-glucan is broken down to smaller fragments and attach themselves to the immune cells which then move effectively throughout the body. It supports the immune system by enhancing the role of innate immunity cells, which then helps eliminate the foreign partilcles like viruses and help the body fight infection or inflammation [43]. An overactive innate immune system is not only a host to the development of autoimmunity but also welcomes the pathogenesis of neurodegenerative diseases, lung fibrosis, inflammatory bowel disease, and in the remodeling process in asthma. The progression and severity of ongoing pandemic disease Covid-19 is due to a dysregulated host immune response resulting in a cytokine storm leading to systemic inflammation and tissue damage [57]. As per clinical data the role of Wellmune β-glucan is to prime the immune response and not just to boost it for it to act imminently to enhance the immune system [58]. Wellmune is effective in improving the functioning of innate immunity by promoting the production of white blood cells specifically leukocytes that is efficient in killing the unwanted pathogens in the body. After the ingestion of wellmune, it binds to specific macrophages and produces biologically active wellmune fragments that enhances the function of neutrophils [59].

#### Prebiotic effect

Cereal β glucan has recently been identified as a well-recognized bioactive carbohydrate with multiple functions and recommended as a potential prebiotic in recent studies. Due to their prebiotic activity, cereal-glucans undergo complete fermentation, which aids gut microbiota for proper digestion [60]. β -glucan enhances the growth of *Prevotella* and *Roseburia* with a consequent increase in the propionate production that results in a positive shift in the microbiome composition and short chain fatty acid production that leads to the maintenance of intestinal barrier integrity, mucus production, and protection against inflammation, and also reduction of the risk of colorectal cancer [61, 62]. β-glucan when administered in a dose-dependent manner was observed to increase the beneficial bacterias that produce short-chain fatty acids such as *Roseburia*, *Ruminococcus*, and *Bifidobacteria* that exude various health benefits while simultaneously decreasing the harmful pathogens [63]. Gut microbiome composition alteration leads to insulin resistance and inflammation. Since β-glucan is degraded in the large intestine and is metabolized by the gut microbiota it significantly decreases the ratio of Firmicutes to Bacteroidetes at the phylum level, it selectively promotes the growth of *Bifidobacterium*, specifically *Bifidobacterium longum* and *Lactobacillus plantarum* [42, 64].

#### Anti-tumor effect

β-glucan exudes antitumorigenic therapeutic benefits. Mushroom β-Glucans emanates immunomodulating actions by stimulating natural killer cells, T-cells, B-cells, neutrophils, and macrophage dependent immune system responses via differing receptors involving dectin-1, the toll-like receptor-2, scavengers and lactosylceramides. It was observed to prevent oncogenesis and even exert anti-tumor activity against allogeneic and syngeneic tumors, and prevent tumor metastasis. The antitumor activity of β-glucan depends on its molecular mass, branching configuration, conformation, and chemical modification. Similarly the mechanism of mushroom β-glucan is mediated by the stimulated T cells or other immune cells leading to various cellular responses such as T cells or other immune cells. Lentinan (a β-glucan extracted from the mushroom Lentinula edodes) enhances the cytotoxicity of peritoneal macrophages against metastatic tumours) [65]. In in vivo studies β-glucan enhanced the host response to various ailments including tumor and showed exceptional results when used in conjunction with chemotherapy [66–70].

#### Antioxidant effect

Oat β-glucan of low molecular weight can transfer hydrogen from molecules that can act as free radical quenchers under physiological conditions. This property is correlated with subduing the oxidative stress in the spleen. The β-glucan produced by *Botryosphaeria rhodina* can exert antioxidant scavenging activities and the anomeric hydrogen in the molecules acts as a free-radical quencher [71, 72]. β-Glucan from barley exerts antioxidant activity that scavenges hydroxyl radicals and is said to have higher activity compared to other polymers [73].

#### Covid-19

β-glucan has immunomodulatory properties, apparently when administered to combat Covid-19, an ongoing global pandemic. Oral β-glucans effectively attenuates the symptoms of COVID-19 and enhances immunity. It induces trained immunity (TRIM) and is considered to be an efficient, low cost and a steady and safe alternative in different age groups with co-existing disease conditions in the defense against COVID-19 [74]. Several responses, such as phagocytosis and/or cytokine secretion, thus activating the innate immune system, can be seen when β-glucan binds to the receptor. The activation of macrophage functions by β-glucans enhances the host's immune defence. When β-glucan binds to Dectin-1, it secretes cytokines such as IL-6, IL-10, IL-12, and TNF-α, which increases the body's response against antigens. β-glucan is capable of activating the complement system through complement system receptor CR3. This receptor is expressed in myeloid cells, NK cells, and lymphocytes. The complement C3 pathway is activated as soon as β-glucan recognizes C3, and this C3b acts directly on macrophages, NK cells, and neutrophils, increasing phagocytosis and lysis of cells that are coated with iC3b (inactivated C3b). These responses affect cells of the immune system, including macrophages, neutrophils, monocytes, NK cells, and DCs [50, 75]. β-glucans improves cellular pattern involved in the infectious process through its immunomodulatory effects. It is also effective in decreasing the cytokines involved in the inflammatory course of COVID-19 [76]. 

#### Glycemic function

Cereal β-glucans can regulate postprandial blood glucose and insulin levels that help prevent diabetes. A study demonstrated that Oat β-glucan effectively decreased insulin and glycemia [77]. Oat β-glucan is effective in decreasing enzyme accessibility, leading to reduced digestion of starch and low postprandial glycaemic response. Oat β-glucans play an influential role as a physical barrier to glucose uptake in absorptive gut epithelial cells IEC-6, thereby affecting the intestinal glucose transport protein1 (SGLT1) and transporter 2 (GLUT2) expression [78]. The gel forming ability of β‐glucan in the small intestine helps slow down the interactions of the digestive enzymes with nutrients and slows down the degradation and absorption of glucose and other nutrients leading a reduced peak postprandial blood glucose concentration for a short period of time [63]. It is also effective in decreasing menaquinol (reduced vitamin K2) biosynthesis, where menaquinol-8 (PWY-5838) was found to be associated with type-2 diabetes [79].

#### Obesity/cholesterol lowering function

Obese children are more likely to get infections and have more severe infections, because of the impaired function of the T cells, a type of white blood cell that specifically helps fight infections and diseases [80]. β-Glucans sourced from plants play a vital role in intercepting high-fat-diet-induced obesity and serum biochemical indicators associated with obesity, fatty liver, and adipocyte size. Barley β-glucan is safe and effectively reduces visceral fat obesity [81]. The action mechanism of dietary fiber in preventing obesity includes: (i) an increase in the viscosity of the intestinal content and a decrease in the energy intake, thereby extending satiety rates and delaying the gastric emptying rate; and (ii) the increase in acetate and butyrate contents in the gut through fermentation which suppresses central appetite and obesity. Thirdly, the SCFAs promotes the release of anorectic gut hormones PYY and GLP-1, thus reducing food intake [82, 83]. β‐glucan has proven to lower cholesterol levels through its gel forming properties. Food and Drug Administration (FDA) has sanctioned the health claim of Oatmeal β‐glucan for lowering the cholesterol serum in cardiovascular diseases [63].

#### Metabolic dysbiosis

Manipulating gut microbiota in the early stages might be a good goal to counter the burgeoning metabolic disorders such as obesity and type 2 diabetes in children. The increase in the β-glucan viscosity inhibits the digestion rate and the fast uptake of the nutrients into the bloodstream, which lowers the glycaemic response, and there is an instant release of insulin, which lowers or increases the rate of bile excretion/lipid absorption, respectively [84, 85].

#### Wound healing

The application of β-glucan-collagen wound dressing matrix onto partial adult wounds reduced pain, improved wound healing, reduced water loss and scab formation, and the number of dressing changes required. Aminated b-(1,3)-d-glucan, a water-soluble Curdlan derivative, stimulated macrophage cell functions such as growth factor and cytokine production in diabetic wounds, resulting in an improved healing response [84].

#### Bowel function

The β-glucan made by *Schizophyllum commune* is effective in the treatment of obesity, obesity related constipation and colitis conditions. Increase in ingestion of fiber resulted in increased defecation frequency, soft stool and quicker transit rate. β-glucan is also effective against inflammatory bowel diseases (IBD), and constipation by ehanching the consistency of the stool and also proliferates goblet cells resulting in a thickened lubricating mucin layer which helps in improving the intestinal transit rate [63, 86]. *Schizophyllum commune* produced β-glucan also has an effect on high-fat diet-induced gut dysbiosis and also reduced obesity-related constipation in mice model [87]. β-glucan also enhances the intestinal motility and also helps recover intestinal microecolgy, thus resulting in an increased neurotransmittance and tight junction protein expression on loperamide-induced constipation [88].

## Importance of β-glucan supplementation in pediatric health

**Fig. 3:**
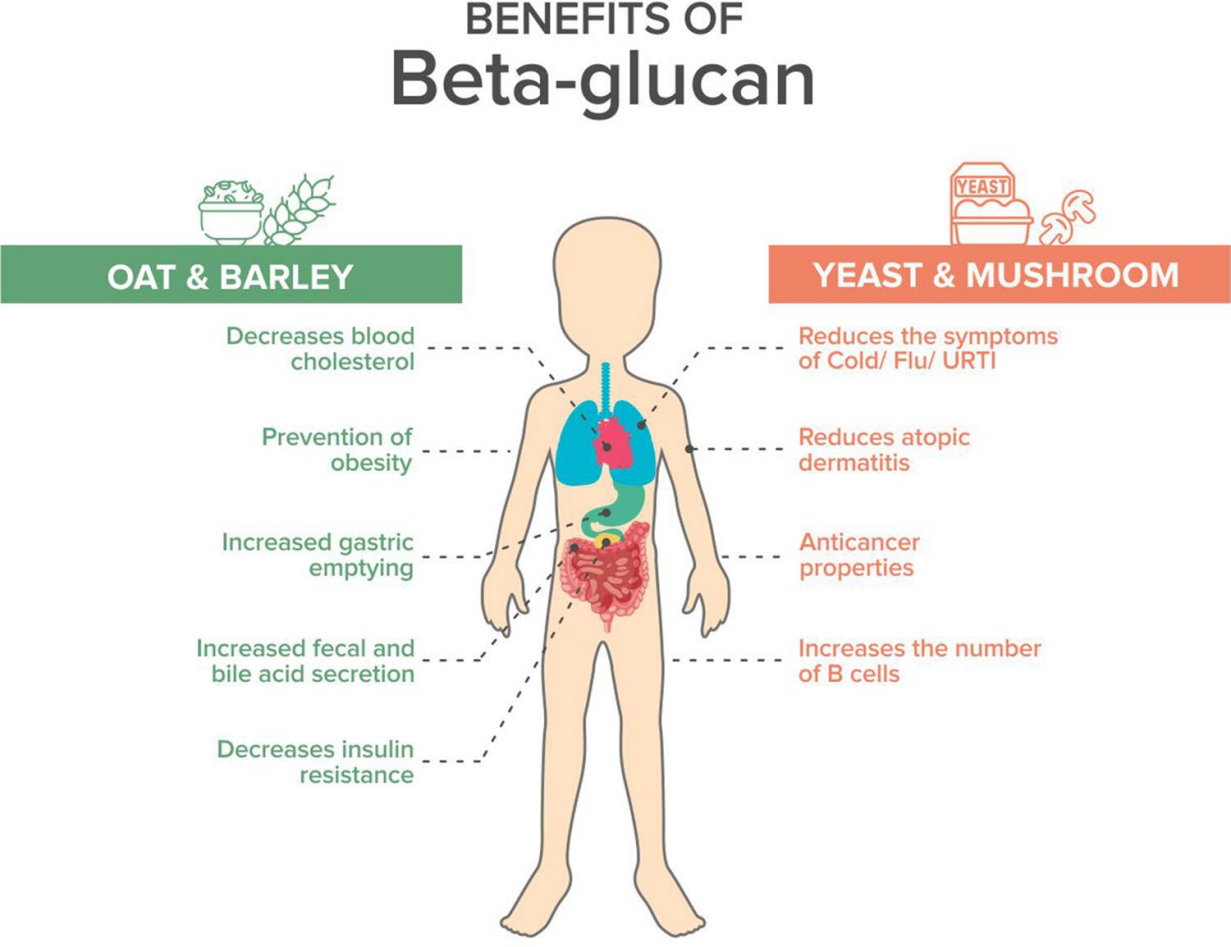
Benefits of β-glucan. Adapted from [47]

The versatile properties of β-glucans include antioxidant properties that scavenge reactive oxygen species; their role as a prebiotic helps prevent cholesterol, and helps production of SCFA. Dietary β-glucans also have immunomodulatory and antitumor properties that activate the mucosal immune system cells [46]. They might lower the risk for heart disease. β-glucans might prevent the body from absorbing cholesterol from food and also stimulate the immune system (Fig. 3).

Lentinan and sonifilan developed from mushrooms have immunomodulatory effects. Medicines derived from β-glucan are administered via injections, although the oral route has shown to be effective in immunomodulatory effects. β-glucan derived from oats and barley has shown excellent serum cholesterol-reducing qualities and prevents metabolic disorders [46].

## Clinical benefits in children

### Respiratory

Children suffering from RRI were given intranasal resveratrol plus carboxymethyl-β-glucan that resulted in reduced nasal obstruction, rhinorrhoea, sneezing, cough, fever, and use of medications [89]. A randomized, double-blind trial was conducted in children suffering from common cold and rhinovirus infection. The nasal spray containing resveratrol plus carboxymethyl-β-glucan might have a positive impact on both clinical and socio-economic burdens due to common cold [90].

A randomized control trial was conducted on children with respiratory infections. The children were fed 3 servings per day of either a FUF with 25 mg DHA, 1.2 g prebiotics polydextrose PDX/galactooligosaccharides GOS, and 8.7 mg yeast β-glucan per serving or an unfortified, cow's milk-based beverage. The results showed that regular consumption of the follow-up formula was associated with fewer episodes and a shorter duration of ARI. Reduced use of antibiotics. It was observed that the intervention group had increased interleukin-10 and white blood cells [91]. A clinical trial was conducted on 37 children with recurrent upper respiratory tract infections during pediatric age. The children were administered an immunostimulant herbal compound of Echinacea Angustifolia, Arabinogalactan, Acerola (Vitamin C), β-Glucan e Zinc (Imoviral® Junior). The results suggest that the herbal component improves the quality of life in pediatric patients. It was also reported that there was a significant improvement in chronic inflammation in the frequency of acute episodes [92].

A randomized clinical trial concluded that colloidal silver and carbossimetyl β-glucan showed a great difference in mean post-treatment Canadian acute respiratory illness and flu scale (CARIFS) global score and CARIFS visual analogue scale (VAS) [93]. A pilot study showed that β-1-3-glucan injections successfully reduced asthamatic symptoms and increased the serum IL-10 levels in children suffering from asthma [94].

A prospective study indicated that nasal congestion, significantly improved after treatment with intranasal β-glucan [95]. The administration of three servings/day of a cow's milk-based beverage containing DHA, the (PDX) and (GOS), β-glucan, and other key nutrients resulted in lower allergic manifestations in the skin and respiratory tract [96].

The short-term supplementation of oral β-glucan (100 mg/day) resulted in the stimulation of physical endurance in children with respiratory problem via stabilization of the sIgA levels, helps their mucosal immunity [97]. In a placebo-controlled, double-blinded, and randomized clinical trial children with allergic rhinitis were treated with resveratrol plus β-glucan or placebo (the diluent of active drug) two sprays (100 µL/spray) in each nostril three times/day for 2 months. There was reduction in nasal symptoms, including itching, sneezing, rhinorrhea, and obstruction significantly in children with pollen induced allergic rhinitis [98]. A double-blind, placebo-controlled, randomised, multicentre study was conducted on children with recurrent respiratory tract infection. The said children were treated with Imunoglukan P4H^®^ syrup (with pleuran-β-glucan from *Pleurotus ostreatus* and vitamin C), or a placebo group (vitamin C only). The syrup was effective in preventing the respiratory tract infections and other symptoms successfully. The syrup also showed immunomodulatory activity [99].

### Immune function

In children with chronic respiratory problems, short-term oral administration of the natural immunomodulator β glucan (100 g) for 30 days significantly increased mucosal immunity [100]. A randomized, double-blinded, placebo-controlled clinical study was performed on asymptomatic children, aged 3–5 years old, the children were given yogurt enriched with β-Glucans from Lingzhi or Reishi Medicinal Mushroom, *Ganoderma lucidum* (Agaricomycetes). It was found that β-glucans from *G. lucidum* increase the frequency of immune system cells in the peripheral blood. A significantly higher absolute count of peripheral blood total lymphocytes (CD3+, CD4+, and CD8+ T cells) was found in the intervention group [101].

### Cognitive function

Children with autism were administered β-glucan food supplements for 90 days. The children had better emotional responses and sleep patterns than the control group. There was a decrease in irritability and anger and improvements in speech characteristics and plasma alpha-synuclein levels [102].

### Glycemic function

A study was conducted on 30 adolescent children with diabetes mellitus type 1 where the children were separated into two groups; each group included natural oat flakes in their regular diet in the quantity of 3 g/day and 6 g/day, respectively for one week. The results of the study demonstrated that minimal glucose levels were the highest, and the pre-meal and post-meal blood glucose levels were lowest for breakfast, lunch, and overall had significantly lower levels of blood glucose in the group consuming 6 g/day of oats [103]. The effect of a β-glucan-enriched bedtime snack was tested on 38 diabetic children for nocturnal blood glucose levels (measured at 2 a.m.). The administration of the β-glucan enriched snack may efficiently flatten the blood glucose levels but cannot prevent nocturnal low blood glucose levels [104].

### Obesity/cholesterol lowering function

A randomized, double-blind, controlled, two-way crossover design study was conducted on children with mild hypercholesterolemia. The children were administered β-glucan containing ready-to-eat cereal that provided 3 g of β-glucan for four weeks. It was found that β-glucan was successful in reducing the LDL cholesterol in subjects with a BMI median of below (25.7 kg/m^2^) [105].

### Sleep patterns

A pilot study was conducted on infants with autism spectrum disorder where the infants were administered yeast-sourced β-glucan, and the average serum melatonin level increased from 238.85 pre-intervention to 394.72 ng/dl post-intervention. Eight out of nine subjects (88%) in the intervention group showed improvement in their sleep pattern and quality. Supplementation of β-glucan extracted from black yeast showed visible improvement in sleep quality, sleep pattern, and serum melatonin levels after ninety days [102].

### Wound healing function

A retrospective chart review was conducted on 225 children with partial thickness burn. The data included children that were administered β-glucan collagen on the debrided wound and secured with sterile strips, kerlix, and an ace wrap. Results indicated that partial thickness wounds are effectively treated in children with β-glucan collagen and BGC markedly simplifies wound care and significantly decreases postinjury pain [106].

### Anticarcinogenic function

A retrospective study was conducted on children with sarcoma. β-glucan was administered as an adjunctive therapy to treat the neutropic fever in these children. The children were administered a nutritional supplement containing 1,3-1,6 β-glucan. The results of the study concluded that the mean length of hospital stay in patients with neutropic fever were higher in the intervention group, although the overall survival rates at 5 years was 83.3% and the event-free survival rates at 5 years was 48.1% in the intervention group whereas in the control group the survival rates and event free rated mounted up to 52.9% and 71% respectively [107].

### COVID-19

A real-world study was conducted on children suffering from a respiratory infection caused due to COVID-19, where the children were administered a food supplement containing Sambucus nigra extract, β-glucan, zinc, and Vitamin D3. The intervention group (food supplement) experienced shorter episodes of respiratory infection compared to the control group, who were subjected to normal treatment for respiratory infection. The food supplement was also successful in reducing respiratory infection episodes in children [108]. A summary of clinical efficacy of β-glucan in children is presented in Tables 1, 2.

**Table 1:** An overview of the clinical efficacy of oral β-glucan in children

Sr. No	Country of study	Condition	Study design	N	Intervention	Duration	Results	References
1	Turkey	Glycemic function	Observational	30 (14–18 years)	standard diet program alone or additional β-glucan 3 g/day or 6 g/day (taken at divided doses of 1 g or 2 g) a two-week washout period between the interventions. Duration—weekly course. Source: Oats	1 week	Phase III had significantly lower levels of SD, CV, LBGI, and CONGA levels than those in either Phase I or Phase II (p < 0.05 for each)	[103]
2	India	Cognitive function	Randomized; parallel group; pilot study	18 (3–18 years)	Group 1 (n = 6): remedial behavioural therapies and L-carnosine 500 mg/day Group 2 (n = 12): β-Glucan (from black yeast) 0.5 g twice a day along with the conventional treatment Source: black Yeast	90 days	• There was a significant decrease in the CARS score in all of the children of the Nichi Glucan Gr.2 compared with the control (p = 0.034517) • Plasma levels of alpha-synuclein were significantly higher in Gr. 2 (Nichi Glucan) than in the control group Gr. 1 (p = 0.091701)	[102]
		Sleep patterns					• In Gr. 2, the average serum melatonin level increased from 238.85 ng/dl pre-intervention to 394.72 ng/dl post-intervention • 8/9 (88%) in Gr. 2 group showed an improvement in their sleep pattern and quality, while this improvement was not observed in the control group	
3	Czech Republic	Innate immunity	Randomized; double blind; placebo-controlled trial	60 (8–12 years)	100 mg/d oral dose of β-glucan Source: yeast	30 days	• A significant increase in the production of lysozyme, CRP, and calprotectin in glucan-treated children • The results were significant at the p < 0.015 level	[100]
4	NA	Cholesterol lowering function	Randomized, double-blind, controlled, two-way crossover design	29 (6–14 years)	Cereal or ready-to-eat cereal containing 3 g/d of β-glucan Source: cereal	4 weeks	• Total and soluble dietary fiber intakes increased significantly (26.7%, p = 0.01 and 30.8%, p = 0.02, respectively) and • LDL cholesterol was reduced by a mean of 5.3% (p = 0.03) • The reduction in LDL cholesterol appeared to be most pronounced (9.2%, p = 0.001) among those with body mass index below the median (25.7 kg/m^2^)	[105]
5	China	Episodes of common cold	Randomized, double-blinded, placebo-controlled study	174 (1–4 years)	75 mg as the high dosage of BYBG and 35 mg as low dosageof BYBG Source: baker's yeast	12 weeks	• Children in either of the two BYBG treatment groups (47% in the BYBG 75 mg/d group, 32% in the BYBG 35 mg/d group) experienced a significantly reduced incidence of infectious illness (p = 0.6314) • The average URTI duration in the Intervention group was less compared to the placebo group by with 3.5 and 2.9 days in the 75 mg/d and 35 mg/d BYBG groups respectively (p < 0.0001) • The average number of URTI episodes in the intervention group was less by 0.7 and 0.5 in the BYBG 75 mg/d and 35 mg/d treatment groups respectively (p < 0.0001)	[109]
6	Italy	URTI	Pilot study	37 (3–10 years)	Herbal compound of Echinacea angustifolia, Arabinogalactan, Acerola (Vitamin C), β-Glucan e Zinc Source: Mushroom	3 months	• After the complete treatment, 77% of children reported an improvement in chronic inflammatory in the frequency of acute episodes • The total score of a questionnaire about life quality is improved (p = 0.04)	[92]
7	Italy	URTI	Randomized clinical trial	100 (0–12 years)	colloidal silver and carbossimetyl β-glucan or placebo (saline solution) Source: NA		• A significant difference in mean post-treatment CARIFS score and CARIFS global VAS in children of group 1 compared with children in group 2 (2.28 ± 1.58 vs. 5.08 ± 3.39; p < 0.001 and VAS: 1.87 ± 1.38 vs. VAS: 3.34 ± 2.19; p = 0.012, respectively) • At the end of treatment, 90% of subjects in group 1 completely recovered, whereas 10% experienced some degree of complications (otitis, tracheitis, bronchitis) • In group 2 a complete recovery was achieved in 66% of subjects, the remaining 34% developed complications	[93]
8	Australia	Blood glucose levels	Pilot study	38 (mean age was 10.8 ± 1.7 years)	A convention bedtime snack consisting of equicaloric β-glucan-enriched food Or Placebo containing an identical amount of carbohydrate Source: NA	228 nights	A significant influence of the type of bedtime snack on the blood glucose course until 2 AM could be observed (p < 0.05)	[104]
9	Brazil	Allergic manifestations in the skin and the respiratory tract	Double-blind, randomized, controlled trial,	256 (1–4 years)	Three servings/day of a cow’s milk-based beverage containing DHA, the prebiotics polydextrose (PDX) and galactooligosaccharides (GOS), β-glucan, and other key nutrients Or A control cow’s milk-based beverage Source: yeast	28 weeks	• The CMBB group had fewer episodes of AM, which included allergic rhinitis or conjunctivitis, wheezing, allergic cough, eczema, and urticaria, compared to the control group (p = 0.021) • The hazard ratio for the increased number of episodes of AM was lower in the CMBB group compared to the control (HR, 0.64; 95% CI 0.47–0.89; p = 0.007)	[96]
10	Czech Republic	Chronic respiratory problems	A randomized, double-blind, placebo-controlled trial	77 (6–15 years)	Oral supplementation of β-glucan 100 mg/day oral dose Source: yeast	4 weeks	In the glucan group, the positive effects of glucan were significant in both age groups. In the glucan group, we found significant decrease of eNO levels and stabilization of the sIgA levels	[97]
11	slovakia	RRI	Double-blind, placebo-controlled, randomised, multicentre study	175 (2–10 years)	Imunoglukan P4H® syrup (with pleuran-β-glucan from *Pleurotus ostreatus* and vitamin C) or Placebo group (vitamin C only) Source: *Pleurotus ostreatus*	12 months	• In the active group, 36% of the children did not suffer from any respiratory infections throughout the treatment, compared to 21% in the placebo group (p < 0.05). • Imunoglukan P4H^®^ also significantly decreased the frequency of flu and flu-like disease and the number of lower respiratory tract infections • Imunoglukan P4H^®^ treatment resulted in a statistically significant modulation of humoral and cellular immunity	[99]
12	China	Respiratory infection	A double-blind, randomized, controlled, prospective trial	39 (3–4 years)	2 servings per day of either a FUF with 25 mg DHA, 1.2 g PDX/GOS, and 8.7 mg yeast β-glucan per serving. (intervention group) Or An unfortified, cow’s milk-based beverage (control group) Source: yeast β-glucan	28 weeks	• The FUF group had fewer episodes and shorter duration of ARI (mean days [SE]; control = 4.3 [0.2]; FUF = 3.5 [0.2]; p = 0.007), less antibiotic use (n [%]; control = 21 [14%]; FUF = 8 [5%]; p = 0.01), and fewer missed days of day care due to illness • The FUF group had higher interleukin-10 and white blood cell count at the end of the study	[91]
13	Colombia	Immune modulation	A randomized, double-blinded, placebo-controlled clinical study	(3–5 years)	Orally administered β-glucans yogurt Source: mushroom	12 weeks	• Children in the group receiving a yogurt with β-glucans presented a significantly higher absolute count of peripheral blood total lymphocytes (CD3+, CD4+, and CD8+ T cells) than that in the group receiving placebo	[101]
14	Italy	Covid-19	Real-world study	298 (2–6 years)	The food supplement containing Sambucus nigra extract, β-glucan, Zinc, and Vitamin D3. was randomly prescribed to children with RRI daily (Active group) The control group were treated with standard therapy for RI Source:Yeast	4 months	• Intervention group had significantly less RI than the Control group, both concerning upper and lower RI (p < 0.001 and 0.003, respectively) during the follow-up period • children in the intervention group experienced shorter RI duration during the treatment and follow-up phases (p < 0.001 for both)	[108]
15	Turkey	Sarcoma (neutropenic fever)	Retrospective study	58 (0–12 years)	A nutritional supplement containing 1,3–1,6 beta-glucan was administered to the intervention group along with the regular chemotherapy patients up to 12 years of age used the syrup form containing pure β-glucan at a dose of 20 mg/day, and patients aged ≥ 12 years used the capsule form containing pure β-glucan at a dose of 50 mg/day Source: Yeast β-glucan	3 years	• The number of patients who had NF and the mean length of hospital stay due to NF were higher in the β-glucan group than those in the control group • The overall survival rates at 5 years were 83.3% and 52.9% and event-free survival rates at 5 years were 48.1% and 71% in the β-glucan and control groups, respectively	[107]

*AM* allergic manifestation, *BYBG* baker’s yeast β-glucan, *CRP* C- reactive protein, *CMBB* cow milk based beverage, *FUF* follow up formula, *GOS* galactooligosaccharide, *LDL* low density lipoprotein, *NF* neutropenic fever, *PDX* prebiotic polydextrose, *RRI* recurrent respiratory tract infection, *sIgA* secretory immunoglobulin, *URTI* upper respiratory tract infection

**Table 2:** Clinical efficacy of other formulations of β-glucan supplementation (other than oral)

Sr. No	Country of study	Condition	Study design	N	Intervention	Duration	Results	References
1	Brazil	Asthma	Open, exploratory study	20 (6–12 years)	First 4 weeks 0.5 mg (0.25 ml) of glucan was given by subcutaneous injections once a week, For the last 4 weeks the interval between injections was increased to every 2 weeks. Source: *Pleurotus ostreatus*	8 weeks	• Mean IL-10 levels were 6.4 pg/ml and 11.3 pg/ml before and after glucan, respectively (p = 0.02) • There was also a reduction of asthmatic symptoms score at the end of the study	[94]
2	USA	Partial thickness	Retrospective chart review	225 (6 weeks–15 years)	BGC was applied to a debrided burn wound and secured with sterile strips, kerlix, and an ace wrap. After 24 h, adherence of the BGC was confirmed and then left open to air. Source:NA	2 years	• Thirty-four patients (79%) had the BGC remain intact while the wound healed underneath, with excellent cosmetic results, minimal analgesic requirements, and no need for repetitive dressing changes	[106]
3	Italy	Nasal obstruction	Prospective study with a pre- and post-design	50 (pediatric patients)	Patients received 2 puffs of β-glucan into each nostril twice a day. Source:NA	4 weeks	• All considered symptoms, including nasal congestion, significantly improved after treatment (p < 0.001)	[95]
4	Italy	Pollen induced allergic rhinitis	Placebo-controlled, double-blinded, and randomized	68 (4–17 years)	Two sprays (100 μl/spray) in each nostril three times/day of Resveratrol plus β-glucan or placebo (the diluent of active drug). Source:Yeast	2 months	• Children treated with active drug achieved a significant reduction in all nasal symptoms: itching (p = 0.0001), sneezing (p = 0.0009), rhinorrhea (p = 0.009), and obstruction (0.002) as well as antihistamine use (p = 0.003)	[98]
5	Italy	Common cold	A randomized double blind clinical study	89 (0–6 months)	A nasal resveratrol/carboxymethyl-β-glucan solution or nasal saline solution. Source:baker’s yeast	30 days	• CARIFS score improved in both groups • Episodes of sneezing and coughing were fewer in the study group after 7 days of follow-up (p < 0.05)	[90]
6	Italy	RRI	Real-life study, randomized	82 (3–12 years)	Resveratrol plus carboxymethyl-β-glucan or saline isotonic solution of (ratio 1:1) Source: Baker’s yeast	90 days	• The active compound was able to significantly reduce the number of days with nasal obstruction, rhinorrhea, sneezing, fever, medication use, medical visits and school absence (all p < 0.001)	[89]

*BGC* β-glucan collagen, *CARIFS VAS* Canadian acute respiratory illness and Flu scale Visual analogue scale, *IL* interleukin, *RRI* recurrent respiratory infection, *URTI* upper respiratory tract infection

## Conclusion

β-Glucans are a group of biologically active fibers or polysaccharides from natural sources with proven medical significance. Clinical benefits of β-Glucan in children have been proven in multiple clinical trials. The diversity of β-glucan makes it versatile in different areas, such as alleviating respiratory symptoms, glycemic control, reducing cholesterol, wound healing, and improving sleep patterns and physical endurance. β-Glucans are known to have antitumor, anti-inflammatory, anti-obesity, anti-allergic, anti-osteoporotic, and immunomodulatory actions. Its application and benefits needs to be further clinically explored and extended to other disease areas like IBS, cancer, bone health etc., in pediatric population which definitely paves way to alternative medicines centred on these polymers.

## Data Availability

Data sharing is not applicable to this article as no datasets were generated or analyzed during the current study.
